# Epigenetic and Tumor Microenvironment for Prognosis of Patients with Gastric Cancer

**DOI:** 10.3390/biom13050736

**Published:** 2023-04-25

**Authors:** Zenghong Wu, Weijun Wang, Kun Zhang, Mengke Fan, Rong Lin

**Affiliations:** Division of Gastroenterology, Union Hospital, Tongji Medical College, Huazhong University of Science and Technology, Wuhan 430074, China

**Keywords:** gastric cancer, tumor microenvironment, epigenetics, machine learning, deep learning, CRISPR

## Abstract

Background: Epigenetics studies heritable or inheritable mechanisms that regulate gene expression rather than altering the DNA sequence. However, no research has investigated the link between TME-related genes (TRGs) and epigenetic-related genes (ERGs) in GC. Methods: A complete review of genomic data was performed to investigate the relationship between the epigenesis tumor microenvironment (TME) and machine learning algorithms in GC. Results: Firstly, TME-related differential expression of genes (DEGs) performed non-negative matrix factorization (NMF) clustering analysis and determined two clusters (C1 and C2). Then, Kaplan–Meier curves for overall survival (OS) and progression-free survival (PFS) rates suggested that cluster C1 predicted a poorer prognosis. The Cox–LASSO regression analysis identified eight hub genes (*SRMS*, *MET*, *OLFML2B*, *KIF24*, *CLDN9*, *RNF43*, *NETO2*, and *PRSS21*) to build the TRG prognostic model and nine hub genes (*TMPO*, *SLC25A15*, *SCRG1*, *ISL1*, *SOD3*, *GAD1*, *LOXL4*, *AKR1C2*, and *MAGEA3*) to build the ERG prognostic model. Additionally, the signature’s area under curve (AUC) values, survival rates, C-index scores, and mean squared error (RMS) curves were evaluated against those of previously published signatures, which revealed that the signature identified in this study performed comparably. Meanwhile, based on the IMvigor210 cohort, a statistically significant difference in OS between immunotherapy and risk scores was observed. It was followed by LASSO regression analysis which identified 17 key DEGs and a support vector machine (SVM) model identified 40 significant DEGs, and based on the Venn diagram, eight co-expression genes (*ENPP6*, *VMP1*, *LY6E*, *SHISA6*, *TMEM158*, *SYT4*, *IL11*, and *KLK8*) were discovered. Conclusion: The study identified some hub genes that could be useful in predicting prognosis and management in GC.

## 1. Introduction

Gastric cancer (GC) is a malignant solid tumor arising from the gastric mucosal epithelium and is the fifth most common tumor and the third leading cause of cancer-related death worldwide [1]. Due to deficient risk classification and preliminary diagnosis of preneoplastic conditions, aggressive cancer behavior, and atrophic gastritis, the five-year survival rate for GC is approximately 20% globally [2,3]. GC is a diverse malignancy caused by various factors, including chronic *Helicobacter pylori* infection, unhealthy diet, Epstein–Barr virus, gene mutation, obesity, and smoking. However, the molecular etiology of GC is yet unknown [4,5]. Chemoradiation and surgical therapy are limited to treating advanced GC patients, and many patients do not respond to the available molecularly targeted medicines. Carcinoembryonic antigen, which includes CA19-9 and carcinoembryonic antigen (CEA), is the most utilized blood biomarker for GC detection, but it lacks specificity and sensitivity [6]. Therefore, there is a pressing need to identify more effective, sensitive, and specific GC biomarkers. More patients have benefited from immunotherapy; for example, pembrolizumab can improve overall survival (OS) with PD-L1 enrichment in patients with PD-L1-positive gastric cancer [7]. Immunotherapy is an additional treatment option for these patients and may be beneficial in the more prevalent clinical regimens.

The tumor microenvironment (TME) includes not only cell types (immune cells, fibroblasts, endothelial cells, etc.), but also extracellular components (hormones, cytokines, extracellular matrix, growth factors, etc.) that surround tumor cells and are supported by a vascular network [8]. The TME is important in tumorigenesis, development, and possibly metastasis, influencing treatment efficacy [9]. Tumor-infiltrating immune cells (TIIC) are drug-targetable cells that influence clinical outcomes by structuring an environment in the TME to limit tumor development and have a potential prognostic value [10]. There is a complex connection with significant prognostic relevance since the immune system performs a dual function by aiding the host barrier and tumor progression [11]. Tumor cells that upregulate immune checkpoint inhibitors (ICIs) with the help of TIICs elude the host immunosurveillance [12]. Epigenetics studies heritable or inheritable mechanisms that regulate gene expression rather than altering the DNA sequence. Epigenetics regulate several biological processes, including cell differentiation, ontogeny, cancer, and development, and is a key mechanism for coupling environmental stress and gene expression [13]. Several epigenetic landscape changes occur in tumor cells, including chromatin modification, DNA hypomethylation at the genomic level [14], dramatically reduced H4K16 acetylation, and other aberrant histone modifications [15]. Epigenetic immune editing has been revealed to promote the acquired immune escape program in the most aggressive subtype of mesenchymal pleomorphic glioblastoma by changing the TME [16]. However, no study has investigated the link between TME-related genes (TRGs) and epigenetic-related genes (ERGs) in GC.

Recently, some machine learning algorithms, such as principal component analysis (PCA), support vector machine (SVM), absolute contraction and selection operator (LASSO), and artificial neural network (ANN), have been demonstrated to effectively improve the problem of increasing statistical errors in high-throughput microarray chips [17]. Few studies on epigenetic and machine learning algorithms exist for developing prediction models and screening candidate genes in cancer samples. Because of its precision, simplicity, and efficiency, CRISPR-Cas9 gene-editing technology has been widely used in the functional investigation of tumor-related genes. A complete review of genomic data was performed to investigate the relationship between epigenesis-TME and machine learning algorithms in GC. Furthermore, putative predictive genes were analyzed in conjunction with cell viability by CRISPR-cas9 screening using the dependency map (DepMap) portal for GC prognosis.

## 2. Materials and Methods

### 2.1. Data Collection

The Cancer Genome Atlas Stomach Adenocarcinoma (TCGA-STAD) and GSE84437 databases were used to obtain RNA-sequence and clinical information. Meanwhile, RNA-sequence information was also downloaded from GSE54129 and GSE65801. The FPKM (fragments per kilobase million) values were transformed to TPM values (transcripts per kilobase million) [18]. The transcriptomic expression data were based on the Illumina HiSeq high-throughput sequencing platform. Meanwhile, batch effects from non-biological technical biases were corrected using the “ComBat” algorithm of “system-verilog-assertions (sva)” R package. The EpiFactors, a comprehensive database of human epigenetic factors and complexes providing 720 ERGs [19] (Table S1), was also used. Table S2 contains 4060 TRGs retrieved from the gene set enrichment analysis (GSEA), MSigDB Team, and earlier publications. Project Achilles is based on a genome-scale CRISPR-Cas9 method for gene knockout to identify genes potentially important for cancer prognosis [20]. The dependency scores for almost 17,000 candidate genes were assessed using the CERES algorithm [21], and important genes with a CERES score <−1 were identified across 75% GC cell lines. The threshold for the differential expression of genes (DEGs) was set at FDR < 0.01 with |log_2_FC| ≥ 1.5. The org. Hs. e.g., db R package was used to convert gene IDs to RNA-seq expression profiles.

### 2.2. Non-Negative Matrix Factorization (NMF) of DEGs in TRGs

The intersection genes were identified using TCGA-STAD and GSE84437 matrices, and their expression levels were determined. NMF is a powerful method for cluster analysis of transcriptome data. It is an effective dimensionality-reduction method that is frequently utilized in the molecular pattern recognition of high-dimensional genomic data. Univariate Cox analysis was used to identify potential TRGs with prognostic relevance. These genes were subjected to NMF cluster analysis using the “NMF” R package. Survival analysis among the detected clusters was done using the “survival” and “survminer” R packages to perform OS and progression-free survival (RFS) analysis. Microenvironment Cell Populations-counter (MCPcounter) is an R program that uses transcriptome data to calculate the absolute abundance of eight immune cells and two stromal cells [22]. Furthermore, changes in immune-cell infiltration were examined in NMF clusters.

Using univariate Cox regression (prognosis-associated genes) and LASSO-penalized regression analyses, a prognostic TME risk score signature was created (independent prognostic risk factors). The data partition was performed using a functional R packet caret (1000 times) to separate 70% of the related TCGA-STAD + GSE84437 expression data into training sets and the remaining 30% into testing sets (Table 1). The risk score for each patient was calculated using the relationship e^sum (gene’s expression × coefficient)^. The median score was used to categorize each sample as low- or high-risk. To determine the prognostic value of our signature, the survival probability using receiver operating characteristic (ROC) curves between different sets was first calculated and then compared. The published signatures (Luo et al. [23], Liu et al. [24], Yu et al. [25], Hu et al. [26], and Shao et al. [27]) were then compared with our TRG signature. Instead of complicated models, a nomogram can count individual numerical estimations of the prognosis probability, and a calibration curve can be used to determine its stability. GSEA was used to analyze the pathway enrichment in the low- and high-risk groups. Additionally, reactions to anti-PD-L1 (atezolizumab) drug therapy were examined in high- and low-risk groups using IMvigor210 cohort [28].

### 2.3. Construction and Validation of the Prognostic ERG Signature

The prognostic value of ERGs was determined using univariate and multivariate Cox regression analyses. The prognostic signature for ERGs was built in the same manner as the signature for TRGs, as stated previously. Based on DEGs, unsupervised cluster analysis (ConsenSuClusterPlus R package) was used to discover distinct epigenetic modification modes and to classify patients for further investigation. The “GSVA” R software was used to perform gene set variation analysis (GSVA) to characterize the biological processes among the epigenetic modification models. For GSVA analysis, the gene set “c2.cp.kegg was used. V6.4. symbols” were obtained from the MSigDB database. The median score was then used to categorize each sample as either low- or high-risk. The “pRRophetic” R package was used to determine the clinical chemotherapeutic response of the high- and low-risk groups. Furthermore, TIMER, XCELL, QUANTISEQ, MCPCOUNTER, EPIC, CIBERSORT-ABS, and CIBERSORT algorithms were employed to generate heatmaps depicting the major component differences of immune cells across the two risk groups. Tumor immune dysfunction and exclusion (TIDE) was also used to evaluate the clinical efficacy of immunotherapy. A higher TIDE prediction score suggests a greater risk of immune evasion; consequently, patients are less likely to benefit from ICI therapy [29]. In addition, a study that performed an extensive immunogenomic analysis of more than 10,000 tumors comprising 33 diverse cancer types by utilizing data compiled by TCGA identified six immune subtypes: wound healing (immune C1), IFN-γ dominant (immune C2), inflammatory (immune C3), lymphocyte depleted (immune C4), immunologically quiet (immune C5), and TGF-β dominant (immune C6) [30].

### 2.4. SVM and ANN Screening for Key Genes

SVM is a supervised learning algorithm that can solve complex classification problems and is based on the structural risk-minimization concept from statistical learning theory [31]. To identify the diagnostic biomarkers of GC, SVM analysis was performed using e1071 R package [32]. A random-forest model for DEGs was built using “randomForest” R package [33]. The top 30 genes with a significance value greater than two for further model development were chosen as disease-specific genes. After normalizing the data to the maximum and lowest values, ANN was used to develop a disease-classification model for the relevant variable using the “neuralnet” R package. The confusion matrix function was used to evaluate the results of five-fold cross-validation to obtain the model accuracy results. The area under the curve (AUC) classification performance verification results were generated using the “pROC” R package [34].

### 2.5. Statistical Analysis

Statistical analysis and results display were performed using R software package (version 3.6.3). Unpaired Student’s t-test and Wilcoxon rank-sum tests were used to determine whether the data were regularly distributed. The chi-squared test determined the relationship between the molecular signature and clinicopathological characteristics. Logistic regression analysis was used to identify independent prognostic factors. The R package “maftools” was used to visualize mutations in the high- and low-risk groups. The cph was utilized and validated using the functions in the “root mean square (RMS)” R package to extract the RMS value of each signature, as well as the hazard ratio (HR) with a 95% confidence interval (CI). The AUC of ROC, C-index score, and RMS curves were used to compare the differences between distinct signatures. DCA was performed on the pooled dataset to determine the clinical effectiveness of this signature. A heatmap was created after unsupervised hierarchical clustering of 30 significant genes in GSE84437, and GSE54129 datasets were categorized using R package “pheatmap”. Statistical significance was defined as *p* < 0.05.

## 3. Results

### 3.1. TME-Related DEGs and NMF Clustering Analysis in TCGA-STAD

**Figure 1:**
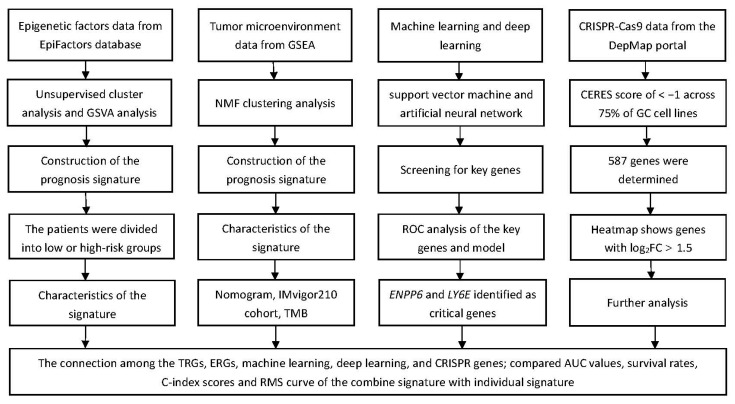
A flow chart of the study.

**Figure 2:**
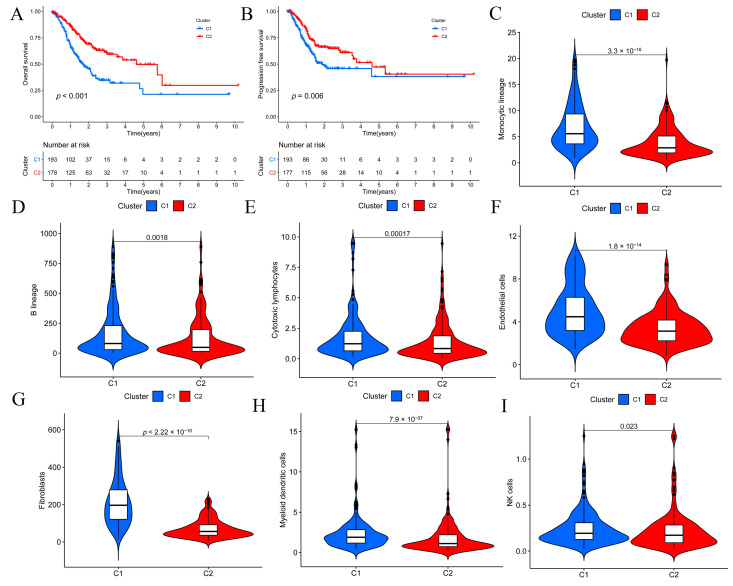
NMF clustering and survival as well as immune analyses across clusters. (**A**) Differences in overall survival (OS) of patients across the two clusters. (**B**) Differences in progression-free survival (PFS) of patients across the two clusters. (**C**–**I**) Differences in immune-cell infiltration between the two clusters; the *Y*-axis represents the absolute abundance of immune cells or stromal cells.

**Figure 3:**
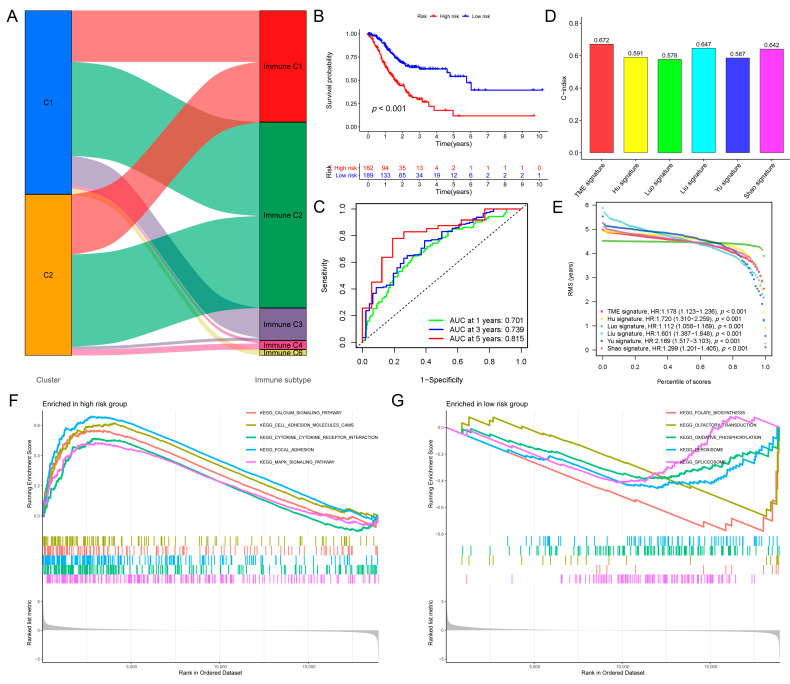
Characteristics of the tumor-microenvironment-related gene signature. (**A**) Results of the Sankey diagram. The results indicated that more immune C3 (inflammatory) and immune C6 (TGF-β dominant) related to C1, more immune C4 (lymphocyte depleted) related to C2. (**B**) Kaplan–Meier survival curves showed that the high-risk group had poorer survival than the low-risk group. (**C**) The AUC values for predicting 1-, 3-, and 5-year survival rates were mostly greater than 0.7, indicating that the model had excellent prognostic predicting value. (**D**) C-index scores of our signature with those from previous published signatures. We found that our signature had comparative performance. (**E**) RMS curve of our signature compared with those from previously published signatures. (**F**) GSEA revealed that the high-risk group was mainly enriched in the MAPK signaling pathway, cytokine–cytokine receptor interaction, focal adhesion, the calcium signaling pathway, and cell adhesion molecules cams. (**G**) GSEA revealed that the low-risk group was mainly enriched in folate biosynthesis, olfactory transduction, oxidative phosphorylation, and spliceosome.

A flow chart of this study is provided in Figure 1, and the clinicopathological characteristics of the patients are illustrated in Table 2. Univariate Cox analysis identified TME-related DEGs with prognostic value based on the TCGA-STAD, and these genes were then subjected to NMF cluster analysis. This study obtained the optimal cluster number (K) using the factoextra package. When K = 2, GC samples were classified into two distinct subtypes (Cluster 1 and Cluster 2), showing a favorable match between GC samples and their identified subtypes (Figures S1 and S2). Kaplan–Meier curves for OS and progression-free survival (PFS) rates suggested that cluster C1 predicted a poorer prognosis (Figure 2A,B). Using the MCPcounter algorithm, an attempt was made to determine the difference in infiltrating immune cells between the two clusters, and the results indicated that C1 was involved in the regulation of infiltration of various immune-cell types, including fibroblasts, endothelial cells, monocytic lineages, and myeloid dendritic cells (Figure 2C–I). The relationship between TRGs and other immunological and molecular subtypes was also explored. Next, TCGA-STAD samples were further categorized according to a pan-patient immune subtype [30] (Table S3). The results indicated that C1 was associated with more immune C3 (inflammatory) and C6 (TGF-β-dominating) cells, while C2 was associated with more immune C4 (lymphocyte deficient) cells (Figure 3A). There was a significant variation in immune-infiltrating cells among clusters, and molecular typing of GC using the NMF model has implications for GC patient prognosis.

### 3.2. Construction and Validation of the Prognostic TRG Signature

**Figure 4:**
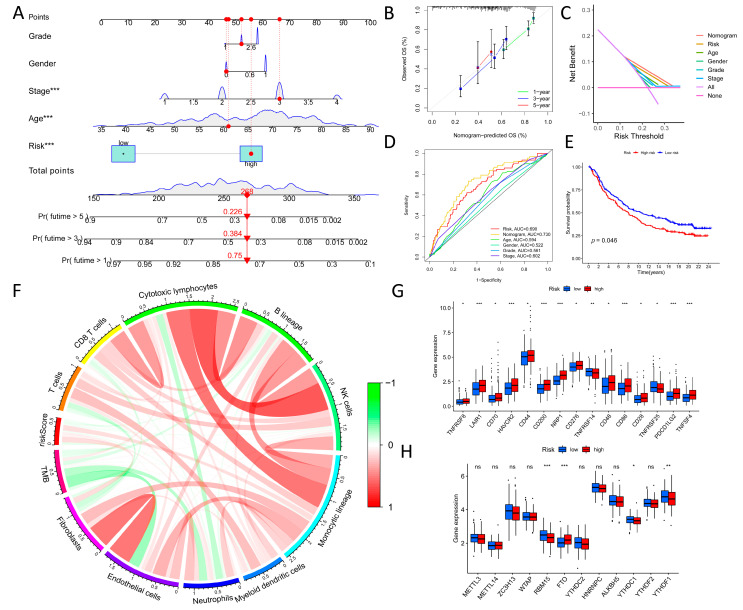
Exploration of tumor-microenvironment-related gene signature and visualization. (**A**) A hybrid nomogram comprising clinicopathological characteristics suggested that the prognostic signature was reliable and stable. (**B**) Calibration curve. (**C**) DCA with clinical traits proved the nomogram’s predictive performance. (**D**) ROC curves proved the nomogram’s predictive performance. (**E**) Overall survival analysis between immunotherapy and risk scores based on the IMvigor210 cohort. (**F**) TMB was negatively correlated with most immune infiltration cells, while risk scores were positively correlated with most immune infiltration cells. (**G**) The expression of immune checkpoint inhibitors (ICIs) among the low- and high-risk groups. (**H**) The expression of m6A-related genes among the low- and high-risk groups; the *Y*-axis represents the relative gene expression based on FPKM. (* *p* < 0.05; ** *p* < 0.01; *** *p* < 0.001; ns, no significant).

After using univariate Cox analysis to identify the potential TRGs with prognostic relevance based on the training sets (70% of the related TCGA-STAD + GSE84437 expression data), Cox–LASSO regression analysis identified eight independent prognostic risk genes (*SRMS*, *MET*, *OLFML2B*, *KIF24*, *CLDN9*, *RNF43*, *NETO2*, and *PRSS21*) to build the prognostic TRG signature (Figure S3A,B). Consequently, a signature was created, and a risk score was produced for each patient to classify them into one of two groups (high- or low-risk) (Table S4). Kaplan–Meier survival curves indicated that the high-risk group fared worse than the low-risk group (Figure 3B). Additionally, AUC values for predicting the 1-, 3-, and 5-year survival rates were typically greater than 0.7, demonstrating that the model had an excellent prognostic, predictive value (Figure 3C). Survival and receiver operating characteristic analyses were performed on the training and testing sets (30% of the related TCGA-STAD + GSE84437 expression data) (Figure S3C–H). The C-index scores of our TME signature (0.672) were the highest when compared to those of the published signatures (Luo signature (0.578), Liu signature (0.647), Yu signature (0.587), Hu signature (0.591), and Shao signature (0.642)) (Figure 3D). Additionally, the signature’s AUC values, survival rates, and RMS curves were evaluated against those of previously published signatures, which revealed that the signature identified in this study performed comparably (Figure 3E and Figure S4). GSEA revealed that the high-risk group was primarily enriched in mitogen-activated protein kinase (MAPK) signaling, cytokine–cytokine receptor interaction, focal adhesion, calcium signaling, and cell adhesion molecules. In contrast, the low-risk group was primarily enriched in folate biosynthesis, olfactory transduction, oxidative phosphorylation, and the spliceosome (Figure 3F,G). Considering the functional similarity between cluster C1 and the high-risk group, it was speculated that the two share some homology, implying poor prognosis. A hybrid nomogram and calibration curve comprising clinicopathological parameters demonstrated that the predictive signature was reliable and stable, implying that it might be used in the clinical care of GC patients (Figure 4A,B). 

Furthermore, decision curve analysis (DCA) with clinical characteristics and results from a mixture of ROC curves validated the nomogram’s predictive ability (Figure 4C,D). Based on the IMvigor210 cohort, a statistically significant difference in OS between immunotherapy and risk scores was observed (Figure 4E). In addition, tumor mutation burden (TMB) was depicted to have a negative correlation with most immune infiltration cells, such as endothelial cells, whereas the TRG signature had a positive correlation with most immune infiltration cells, such as fibroblasts and monocytic lineage cells, which means that our signature was correlated with TME (Figure 4F). Furthermore, the expression of immunological checkpoints (ICIs) and N6-methyladenosine (m6A)-related genes across the two groups was compared, which indicated that the expression of *CD44*, *CD276*, *RBM15*, *FTO*, *YTHDC1*, and *YTHDF1* was significantly different between the high- and low-risk groups (Figure 4G,H). In summary, the signature identified in this study can provide additional evidence that the clinical prediction of GC patients can be improved.

### 3.3. Construction and Validation of the Prognostic ERG Signature

**Figure 5:**
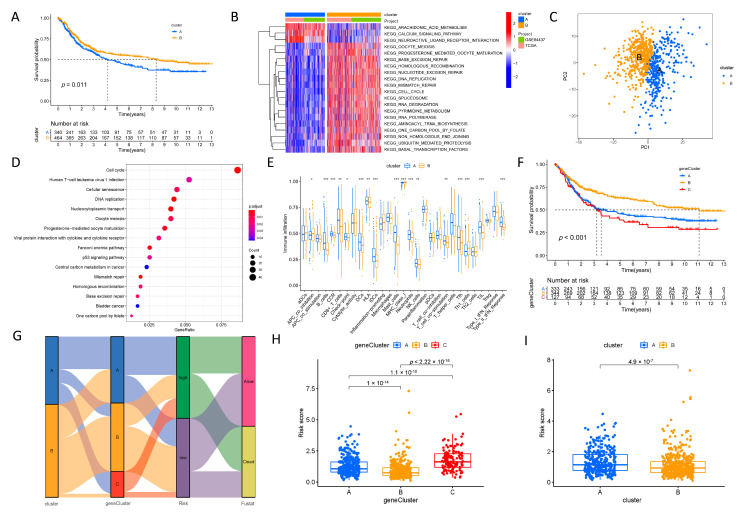
Construction and validation of the prognostic epigenetic-related gene signature. (**A**) Cluster A was related to poor survival. (**B**) GSVA enrichment analysis indicated that cluster A was mainly enriched in arachidonic acid metabolism, the calcium signaling pathway, and neuroactive ligand receptor interaction; cluster-B was associated with nucleotide excision damages and repairs such as spliceosome and homologous recombination. (**C**) Principal component analysis (PCA) of the transcriptome profiles in the two clusters. (**D**) The KEGG analysis showed that the co-expressed genes were mainly enriched in pathways related to cancers and the cell cycle. (**E**) Immune-cell differences among the two clusters based on the ssGSEA algorithm. (**F**) Unsupervised cluster analysis based on the 928 genes was used to classify patients into three gene clusters A/B/C and gene cluster C was related to the worst prognosis. (**G**) Ggalluvial diagram to visualize the connection among the epigenetic clusters, gene clusters, and risk groups. (**H**) The connection between the risk scores and gene clusters. (**I**) The connection between the risk scores and clusters. (* *p* < 0.05; ** *p* < 0.01; *** *p* < 0.001; ns, no significant).

First, two distinct groups (A and B) were identified using unsupervised clustering, with cluster A being associated with low survival based on TCGA-STAD + GSE84437 (Figure 5A). GSVA enrichment analysis revealed that cluster A was primarily enriched in arachidonic acid metabolism, the calcium signaling pathway, and neuroactive ligand–receptor interaction. In contrast, cluster B was linked to nucleotide excision damage and repair, such as splicing and homologous recombination (Figure 5B). Figure 5C displays the principal component analysis (PCA) of the transcriptome profiles of the two clusters, which were found to have significant transcriptome differences. DEGs in each cluster were detected, and 928 co-expressed genes were identified. KEGG analysis indicated that the co-expressed genes were primarily enriched in cancer and cell-cycle pathways (Figure 5D). The ssGSEA algorithm was then used to compare immune-cell differences between the two groups. The findings revealed that cluster A was associated with most immune cells, implying that cluster A may contribute to tumor immunity (Figure 5E). These findings suggest that epigenetic changes are linked to tumor growth. The 928 genes were then utilized in an unsupervised cluster analysis to classify patients into three gene clusters, A/B/C, with gene cluster C being associated with a worse prognosis (Figure 5F). The prognostic signature was built using Cox–LASSO regression analysis, which identified nine genes (*TMPO*, *SLC25A15*, *SCRG1*, *ISL1*, *SOD3*, *GAD1*, *LOXL4*, *AKR1C2*, and *MAGEA3*) as independent prognostic genes (Table S5). As a result, a signature was created, and a risk score for each patient was generated by dividing the patients into high- and low-risk groups. Furthermore, a ggalluvial diagram depicts the relationship between epigenetic clusters, gene clusters, and risk groups. The findings demonstrated that cluster A (determined by 4060 TRGs based on the unsupervised clustering method) had a clear interrelationship with cluster C (determined by 928 DEGs based on the unsupervised clustering method), implying that the prognostic classification of this study was reasonably accurate (Figure 5G–I).

**Figure 6:**
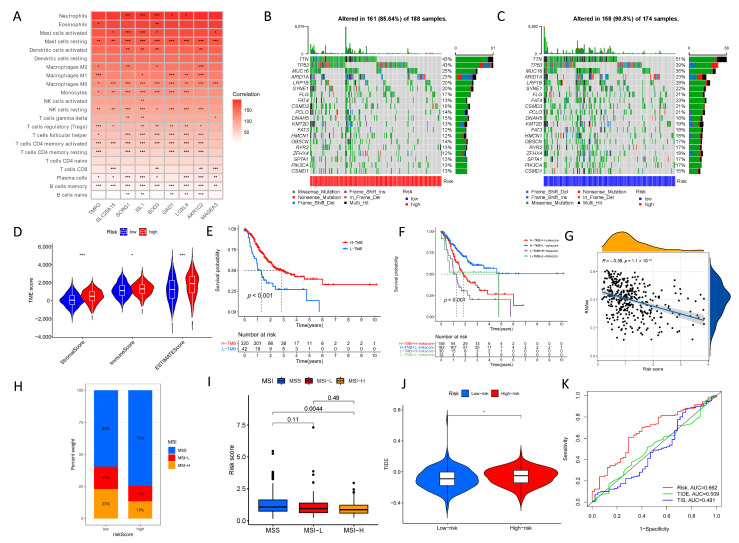
Characteristics of the epigenetic-related gene (ERG) signature. (**A**) The correlation among the identified nine genes and the immune cells based on the CIBERSORT algorithm. (**B**) The differences in somatic mutations in the high-risk groups. (**C**) The differences in somatic mutations in the low-risk groups. (**D**) The stromal score, immune score, and ESTIMATE score was also significance between the two risk groups based on the estimate TME algorithm. (**E**) The high-TMB group had a poorer prognosis. (**F**) Low TMB combined with a high-risk score was also related to poor outcomes. (**G**) The risk score had a negative correlation with the stemness. (**H**) The high-risk group contained more microsatellite stability (MSS) events. (**I**) There was a statistical difference between the MSS and MSI-high groups. (**J**) Patients with high ERGs had a higher TIDE score. (**K**) The risk signature was superior to the 18-gene T-cell-inflamed signature (TIS) and TIDE models. (* *p* < 0.05; ** *p* < 0.01; *** *p* < 0.001; ns, no significant).

Meanwhile, survival and ROC analyses were performed in both the training and testing sets, where the high-risk group had a lower survival rate than the low-risk group (Figure S5A–F). Using the CIBERSORT algorithm [35], the relationship between the selected nine genes and immune cells was examined. Figure 6A displays that these genes were linked with most immune-infiltrating cells. Variations in somatic mutations between the low- and high-risk groups were also investigated, and *TTN* was the most frequently mutated gene (Figure 6B,C). Based on the estimated TME algorithm, the stromal, immune, and ESTIMATE scores were likewise significant between the two risk groups (Figure 6D).

**Figure 7:**
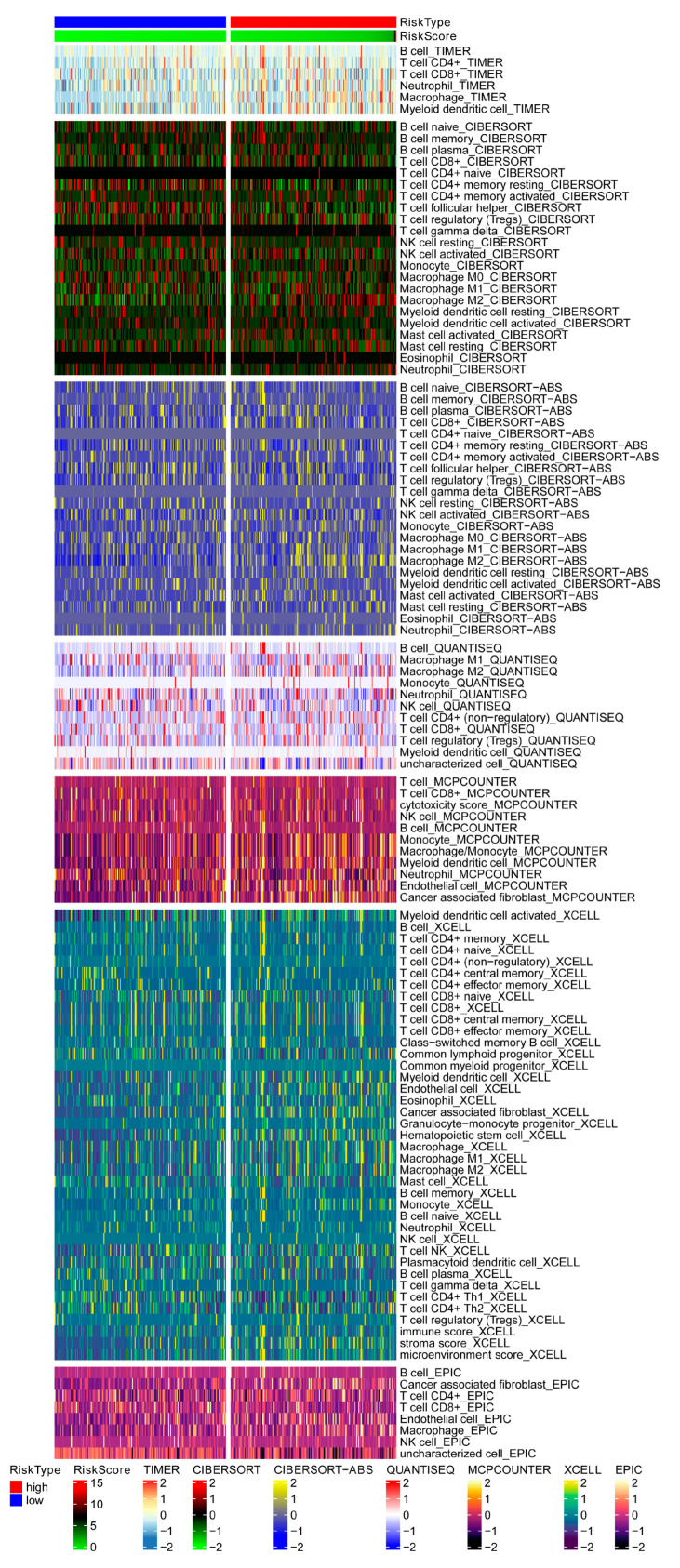
Heatmap showing immune responses based on different algorithms.

Furthermore, the high TMB group had a worse prognosis, and when low TMB was paired with a high-risk score, it was also associated with poor results (Figure 6E,F), indicating that the high TMB group might predict the efficacy of immunotherapy. Stemness is a major cause of tumor recurrence and medication resistance [36], and it was discovered that the risk score had a negative correlation with stemness (Figure 6G). Meanwhile, the study discovered that the low-risk group had more MSI-low and -high events. In contrast, the high-risk group had more microsatellite-stability (MSS) events, with a statistically significant difference between the MSS and MSI-high groups (Figure 6H,I). In this study, patients with high ERGs had a higher TIDE score, and the predictive value of the risk signature outperformed the 18-gene T-cell-inflamed signature (TIS) and TIDE models [37,38] (Figure 6J,K). Figure 7 depicts a heatmap of immunological responses based on several algorithms. Overall, the ERG signature in this study provided the expected predictive performance for GC prognosis. Additionally, a high-risk score was associated with IC_50_ of chemotherapeutics such as lenalidomide, metformin, and pyrimethamine, implying that the signature might be a prospective predictor of chemosensitivity (Figure S5G–I).

### 3.4. SVM and ANN Screening for Key Genes

**Figure 8:**
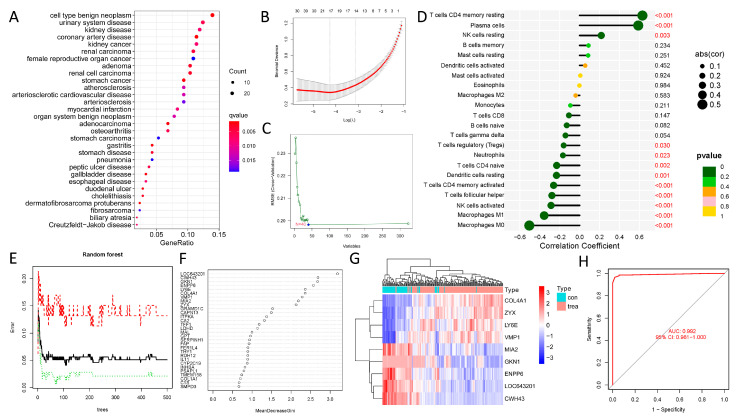
SVM and ANN screening for key genes. (**A**) Disease ontology (DO) analysis revealed that these DEGs were mainly enriched in cancers, such as GC. (**B**) LASSO regression analysis identified 17 key DEGs. (**C**) The SVM model identified 40 key DEGs. (**D**) The correlation between the ENPP6 and immune cells based on the CIBERSORT algorithm. (**E**) The connection plot of the model error and the number of decision trees. (**F**) Nine DEGs with an importance greater than 2 as the key genes. (**G**) The heatmap of the 9 DEGs. (**H**) The neural network model based on the 9 DEGs; the AUC result was 0.992, which displays the robustness of the model.

After data normalization, GSE54129 and GSE65801 gene matrix data were combined for the next analysis. After analyzing the gene-expression profile, which contained 323 DEGs, 123 were upregulated, and 200 were downregulated. Figure 8A depicts the disease ontology (DO)-analysis results, showing that these DEGs were primarily enriched in malignancies, such as GC. It was followed by LASSO regression analysis to identify 17 key DEGs and an SVM model to identify 40 significant DEGs, and based on the Venn diagram, eight co-expression genes (*ENPP6*, *VMP1*, *LY6E*, *SHISA6*, *TMEM158*, *SYT4*, *IL11*, and *KLK8*) were discovered (Figure 8B,C). According to ROC results, AUC value for all genes was >0.86, with *ENPP6* having the highest AUC value of 0.957. Figure S6 shows that the main gene risk classifier demonstrated good discrimination between GC and normal samples with high specificity and sensitivity for GC diagnosis. The relationship between *ENPP6* and immune cells was also investigated using the CIBERSORT algorithm (Figure 8D). A total of 323 DEGs were entered into a recurrent random-forest classification for all feasible numbers inside the variables, and the average error-rate of the model was determined. Figure 8E depicts the relationship between the model error and the number of decision trees. Then, nine DEGs (*LOC643201*, *CWH43*, *GKN1*, *ENPP6*, *LY6E*, *COL4A1*, *VMP1*, *MIA2*, and *ZYX*) were determined with a significance greater than two as the key genes for further investigation, with *LOC643201* being the most significant (Figure 8F). The tumor and normal samples were discriminated by these nine critical variables (Figure 8G), constructing a neural network model based on the nine DEGs and the AUC value was 0.992, indicating the robustness of the model (Figure 8H). Interestingly, *ENPP6* and *LY6E* were identified as important genes using SVM and ANN deep-learning techniques.

### 3.5. Identification of Essential Genes by CRISPR 

**Figure 9:**
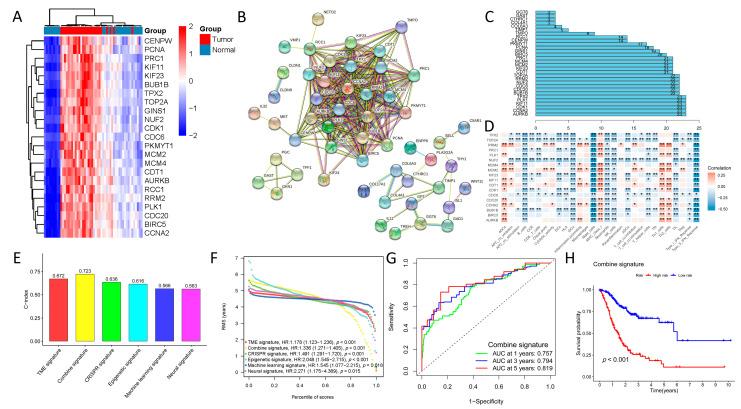
The connection among the tumor-microenvironment-related, epigenetic-related, deep-learning, and CRISPR genes. (**A**) The heatmap shows genes with log_2_FC > 1.5 based on the DepMap. (**B**) Protein–protein interaction (PPI) network analysis. (**C**) Five hub genes (*TPX2*, *PLK1*, *CDK1*, *CCNA2*, and *AURKB*) was identified. (**D**) The correlation with the immune-infiltrating cells. (**E**) C-index scores of the combined signature with individual signatures. (**F**) RMS curve of the combined signature with individual signatures. (**G**) AUC values of the combined signature with individual signatures. (**H**) Survival rates of the combined signature with individual signatures. (* *p* < 0.05; ** *p* < 0.01; ns, no significant).

CRISPR-based genome-wide loss-of-function screening was performed to identify the critical genes generated by DepMap. A total of 587 genes were identified as being critical for the prognosis of GC cell lines. Table S6 and the heatmap in Figure 9A reveal genes with log_2_FC > 1.5 and FDR < 0.01. This was followed by analyzing the relationships between TRGs, ERGs, deep-learning, and CRISPR genes. After performing a protein–protein interaction (PPI) network analysis on all these genes and counting the number of interactions for each, five hub genes (*TPX2, PLK1, CDK1, CCNA2,* and *AURKB*) were identified (Figure 9B,C). The correlation with immune-infiltrating cells was also investigated in Figure 9D, and the relationship with immune-infiltrating cells was also studied (Figure 9D). Finally, a combined signature based on TRGs, ERGs, deep-learning, and CRISPR genes was built and compared to individual signatures and the comparative performance of the combined signature was determined (Figure 9E–H and Figure S7). Overall, the prognostic risk signatures built from multi-omics data were relatively accurate, with reduced heterogeneity, and could effectively differentiate the prognosis of GC patients.

### 3.6. Expression of Prognostic Differentially Expressed Genes

**Figure 10:**
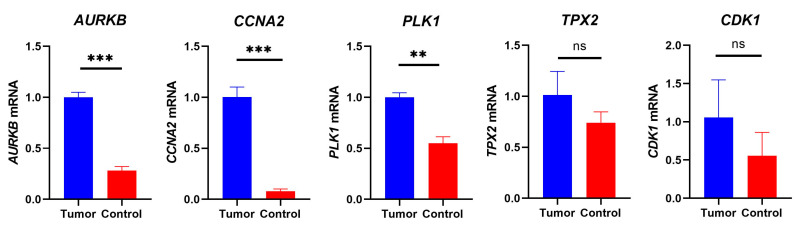
The mRNA levels of *AURKB*, *CCNA2*, *PLK1*, *TPX2*, and *CDK1* were measured by RT-PCR. (** *p* < 0.01; *** *p* < 0.001; ns, no significant).

**Figure 11:**
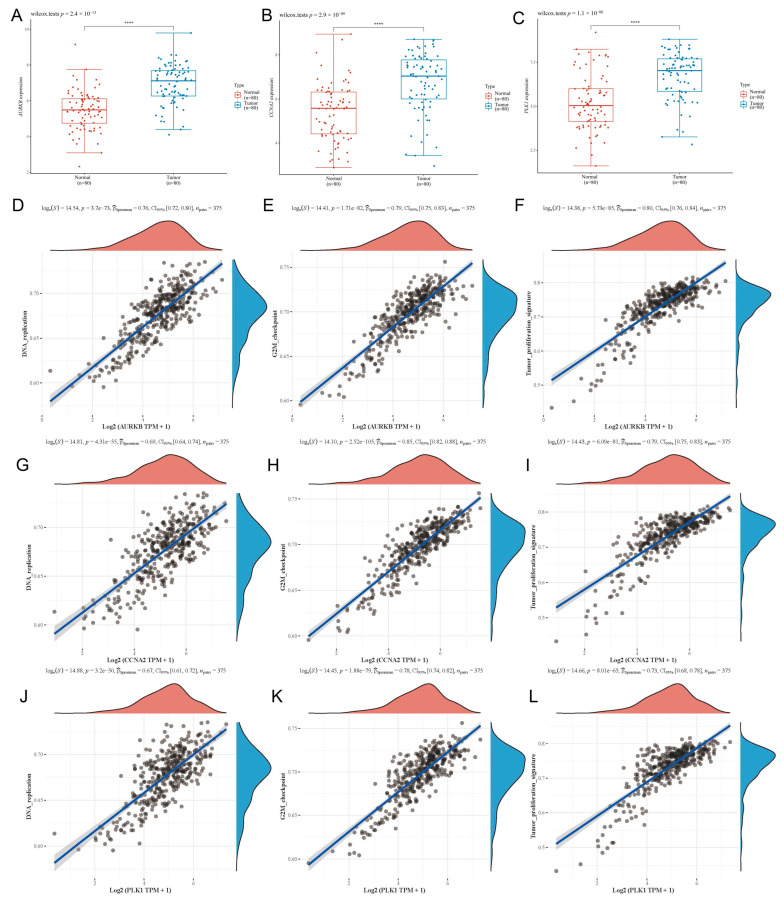
The expression of the *AURKB*, *CCNA2*, and *PLK1* in the GSE27342 dataset. (**A**) The expression of *AURKB*. (**B**) The expression of *CCNA2*. (**C**) The expression of *PLK1*. (**D**–**F**). The correlation between the *AURKB* and DNA replication, G2M checkpoint, and tumor proliferation signature pathways. (**G**–**I**). The correlation between the *CCNA2* and DNA replication, G2M checkpoint, and tumor proliferation. (**J**–**L**) The correlation between the *PLK1* and DNA replication, G2M checkpoint, and tumor proliferation. **** *p* < 0.0001.

We examined the protein levels of these eight genes in risk models using the Human Protein Atlas (HPA) database. The results demonstrated that GC tissues had higher protein levels for most genes (Figure S8). Previous studies have reported the differential expression of the genes we identified in gastric cancer, but few studies have reported the differential expression of *AURKB*, *CCNA2*, *PLK1*, *TPX2*, and *CDK1* in gastric cancer. As expected, *AURKB*, *CCNA2*, and *PLK1* were upregulated in the tumor cell line compared to that in normal gastric epithelial cells (Figure 10). Next, in order to verify the expression of the *AURKB, CCNA2*, and *PLK1* in clinical gastric tissues, we used the GSE27342 as the validation dataset. The results revealed the high expression of *AURKB*, *CCNA2*, and *PLK1* in tumor tissues when compared to normal tissues and we also found that there was a strong positive correlation with the DNA replication, G2M checkpoint, and tumor proliferation signature pathways (Figure 11). 

## 4. Discussion

Epigenetic modifications can govern gene activity without modifying the DNA-sequence basis, thereby influencing tumor evolution and the genesis and development of heterogeneity. These changes and microenvironmental factors eventually mediate the clinical features of precancers and malignancies and can be used as biomarkers for tumor risk stratification. Epigenetic regulatory medications are likely to substantially impact the TME by encouraging transcription and metabolic reprogramming in local immune-cell populations, thereby inhibiting immunosuppressive cells and activating anti-cancer T effector cells. We have previously explored the role of ERGs and inflammatory-response-related gene signatures in hepatocellular carcinoma [39], but the role of TRG signatures and ERGs in GC was not explored by combining NMF clustering, CRISPR-based, and deep-learning analysis. Immunotherapy response was also analyzed using IMvigor210 cohort and TIDE models, and more importantly, we also applied SVM and ANN screening for key genes in GC. Accordingly, in this study, we first created a TRG and ERG signature, then identified several hub genes, and employed various approaches to investigate the relationships between these genes in GC patients. These findings may contribute to a better understanding of epigenetic and TME changes in GC, as well as possible biomarkers for clinical therapeutic intervention. 

During modeling, eight genes (*SRMS*, *MET*, *OLFML2B*, *KIF24*, *CLDN9*, *RNF43*, *NETO2*, and *PRSS21*) have been shown to play essential roles in various types of malignancies in multiple studies. Src-related kinase is a non-receptor tyrosine kinase that lacks C-terminal regulatory tyrosine and N-terminal myristoylation sites (*SRMS*) [40]. Based on proteomic analysis of serum samples, Yoo et al. [41] found that *SRMS* were the only kinases differentially expressed in GC compared with normal controls. The *SRMS* SH2 domain demonstrated significant binding [42]. *MET* is a tyrosine kinase receptor for hepatocyte growth factor (HGF) and is encoded by the proto-oncogene, c-Met. Palle et al. [43] revealed that HGF indirectly induces Treg accumulation in the peripheral blood of GC patients via c-Met-expressing monocytes. Meanwhile, GC with many stromal cells and low *MET* expression may benefit more from MET-targeted therapies. Olfactomedin-like 2 B (*OLFML2B*) is an extracellular matrix protein comprising an olfactomedin (OLF) domain and a region upstream of the OLF domain rich in Ser/Thr residues [44]. *OLFML2B* overexpression was associated with a worse prognosis in GC, and OLFML2B knockdown lowered the migration and proliferation abilities of bladder cancer cell lines [45,46]. *KIF24*, a microtubule-depolymerizing kinesin that localizes preferentially to mother centrioles, is phosphorylated by Nek2, enhancing its activity and preventing cilia from outgrowing in proliferating cells [47,48]. Claudin-9 (*CLDN9*)-overexpression enhances the tumorigenic features of a GC cell line [49] and affects the STAT3 signaling pathway via Tyk2 to boost hepatocyte metastatic ability [50]. Ring finger protein 43 (*RNF43*) expression is decreased in recurrent GC, and loss of *RNF43* activity confers resistance to DNA-damaging radiation and chemotherapy in gastric cells [51]. Overexpression of neuropilin and tolloid-like 2 (*NETO2*) promotes GC cell invasion and migration in vitro and metastasis in vivo, consistent with promoting epithelial–mesenchymal transition [52]. Testisin is a glycosyl-phosphatidylinositol-linked serine protease that is encoded by *PRSS21*. In soft agar, silencing endogenous testis in mRNA results in increased apoptosis and decreased growth [53]. The TRGs identified in this study may play a role in carcinogenesis and require further investigation.

By increasing the production of tumor-related antigens through transcriptional inhibition, epigenetic regulators can coordinate and increase tumor immunogenicity [54]. Consequently, the prognostic ERG signature was generated in this work, nine genes were identified as independent prognostic genes, and their possible association with TME was investigated. Deep learning has made tremendous strides in cancer research and is superior to standard machine-learning methods [55]. ANNs are frequently used for cancer diagnosis and survival analysis [56]. *ENPP6* and *LY6E* were identified as important genes in this study using SVM and ANN deep learning. *ENPP6* is a phospholipase C that synthesizes phosphocholine from choline-containing lysophospholipids [57]. Asundi et al. [58] discovered that *LY6E* was highly expressed and amplified in a diverse array of human solid tumors. Dendritic cells (DCs) loaded with the *LY6E* peptide antigen can initiate and boost murine T-cell proliferation [59]. Furthermore, LY6E siRNA induced apoptosis and G1-S cell-cycle arrest [60]. Five hub genes (*TPX2*, *PLK1*, *CDK1*, *CCNA2*, and *AURKB*) were also identified based on PPI analysis. According to a meta-analysis, increased expression of the targeting protein for Xenopus kinesin-like protein 2 (*TPX2*) is associated with poor OS in gastrointestinal-tract cancers [61], old age, and tumor T stage in GC [62]. Polo-like kinase 1 (*PLK1*) inhibitors, si-PLK1 and BI2536, may restore chemosensitivity in drug-resistant SGC-7901/cisplatin cells and increase cisplatin efficacy [63]. CDK1 phosphorylates ISL1 on serine 269 and increases its binding to cyclin B2 and cyclin B1 promoters and its transcriptional activity in GC [64]. Cyclin A2 (*CCNA2*) expression is increased in KRAS mutant GC cell lines and primary tumors, leading to increased susceptibility to *PLK1* inhibitors [65]. Downregulation of aurora kinase B (*AURKB*) decreases GC cell proliferation, promotes apoptosis, halts the cell cycle in G_2_/M phase, and suppresses GC cell migration and invasion [66]. When our constructed signatures and hub genes are combined with current indicators, predicting the prognosis of GC patients may be more accurate and efficient, which has clinical benefits in tumor care. There were a few drawbacks to this study. Even though we completed many verifications across multiple databases to obtain an objective and comprehensive evaluation, the built signature still lacks clinical validation based on distinct cohorts; hence, more clinical trials are required in the future. Furthermore, the primary mechanism underlying the clinical success of the model is unknown, and this study used a retrospective approach, which could have resulted in bias.

## 5. Conclusions

In this study, we initially created a profile of TME-related and epigenetic-related genes and identified certain hub genes. Various approaches have investigated the relationship between these genes in GC patients. These findings could be useful for predicting the prognosis and management of GC.

## Figures and Tables

**Table 1:** The clinicopathological characteristics in the training set and testing set.

Covariates	Type	Total	Test	Train	*p* Value
Age	<=65	163 (43.94%)	46 (42.59%)	117 (44.49%)	0.8363
	>65	205 (55.26%)	61 (56.48%)	144 (54.75%)	
Gender	Female	133 (35.85%)	38 (35.19%)	95 (36.12%)	0.9588
	Male	238 (64.15%)	70 (64.81%)	168 (63.88%)	
Grade	G1	10 (2.7%)	2 (1.85%)	8 (3.04%)	0.1032
	G2	134 (36.12%)	48 (44.44%)	86 (32.7%)	
	G3	218 (58.76%)	56 (51.85%)	162 (61.6%)	
Stage	Stage I	50 (13.48%)	12 (11.11%)	38 (14.45%)	0.0967
	Stage II	111 (29.92%)	38 (35.19%)	73 (27.76%)	
	Stage III	149 (40.16%)	35 (32.41%)	114 (43.35%)	
	Stage IV	38 (10.24%)	15 (13.89%)	23 (8.75%)	
T	T1	18 (4.85%)	4 (3.7%)	14 (5.32%)	0.043
	T2	78 (21.02%)	19 (17.59%)	59 (22.43%)	
	T3	167 (45.01%)	61 (56.48%)	106 (40.3%)	
	T4	100 (26.95%)	22 (20.37%)	78 (29.66%)	
M	M0	328 (88.41%)	90 (83.33%)	238 (90.49%)	0.0503
	M1	25 (6.74%)	12 (11.11%)	13 (4.94%)	
N	N0	108 (29.11%)	37 (34.26%)	71 (27%)	0.5256
	N1	97 (26.15%)	28 (25.93%)	69 (26.24%)	
	N2	74 (19.95%)	19 (17.59%)	55 (20.91%)	
	N3	74 (19.95%)	19 (17.59%)	55 (20.91%)	

**Table 2:** The clinicopathological characteristics of GC patients.

Clinical Characteristics	TCGA (N = 443)	GSE84437 (N = 433)
Age at diagnosis (y)	65 (30–90)	60 (27–86)
Survival time (day)	565 (0–3720)	2100 (0–4830)
Gender		
Female/Male	158/285	137/296
Stage		
I/II/III/IV/NA	59/130/183/44/27	NA
Grade		
G1/G2/G3/GX	12/159/262/13/5	NA
T-classification		
T1/T2/T3/T4/TX	23/93/198/119/10	11/38/92/292
M-classification		
M0/M1/MX	391/30/22	NA
N-classification		
N0/N1/N2/N3/NX	132/119/85/88/19	80/188/132/33
Status		
Alive/Death	272/171	224/209

Data express as Mean (min–max). NA: not applicable.

## Data Availability

All data were downloaded from public databases, containing The Cancer Genome Atlas (TCGA, https://tcga-data.nci.nih.gov/tcga/, accessed on 18 September 2022) and the Gene Expression Omnibus (GEO, http://www.ncbi.nlm.nih.gov/geo/, accessed on 20 September 2022).

## Supplementary Materials

The following supporting information can be downloaded at: https://www.mdpi.com/article/10.3390/biom13050736/s1: Figure S1: Tumor-microenvironment-related differential expression of genes performed NMF clustering analysis and determined two clusters (C1 and C2). Figure S2: Heatmap of different clusters based on the NMF clustering analysis. Figure S3: Construction and validation of the prognostic tumor-microenvironment-related gene signatures. (A) λ where the smallest partial likelihood deviance. (B) Results of the curve fitting. (C) Survival analysis in the TCGA training set. (D) Survival analysis in the TCGA testing set. (E) Survival analysis in the GSE84437 set. (F) ROC analysis in the TCGA training set. (G) ROC analysis in the TCGA testing set. (H) ROC analysis in the GSE84437 set. Figure S4: AUC values and survival rates of the signature with those from previous published signatures (namely by Luo et al. [23], Liu et al. [24], Yu et al. [25], Hu et al. [26], and Shao et al. [27]). Figure S5: Construction and validation of the prognostic epigenetic-related gene signatures. (A) Survival analysis in the TCGA all set. (B) Survival analysis in the TCGA training set. (C) Survival analysis in the TCGA testing set. (D) ROC analysis in the TCGA all set. (E) ROC analysis in the TCGA training set. (F) ROC analysis in the TCGA testing set. (G) High-risk score was related to an IC50 of chemotherapeutics such as pyrimethamine. (H) Lenalidomide. (I) Metformin. Figure S6: The ROC results suggested that AUC values for all gene were >0.86 and *ENPP6* with maximum AUC value 0.957. Figure S7: AUC values and survival rates of the combine signature with individual signature. Figure S8: Immunohistochemistry in the normal and tumor groups from the Human Protein Atlas database. Table S1: The 720 epigenetic-related genes obtained from the EpiFactors database. Table S2: Tumor-microenvironment-related to genes were extracted from the gene set enrichment analysis (GSEA) MSigDB Team and previous publications. Table S3: The 355 immune samples classified according to pan-patient immune subtypes. Table S4: Cox–LASSO regression analysis was utilized to construct the prognostic model, and screen out 8 genes. Table S5: Cox–LASSO regression analysis construct the prognostic signature, and identified 9 genes as independent prognostic genes. Table S6: The 587 genes that were determined as crucial in the prognosis of gastric cancer cell lines based on the DepMap.
