# Supplementary figures and images for: Epigenetic and Tumor Microenvironment for Prognosis of Patients with Gastric Cancer
Source: Biomolecules. 2023 Apr 25;13(5):736. doi: 10.3390/biom13050736 (PMC10216680; doi:10.3390/biom13050736)

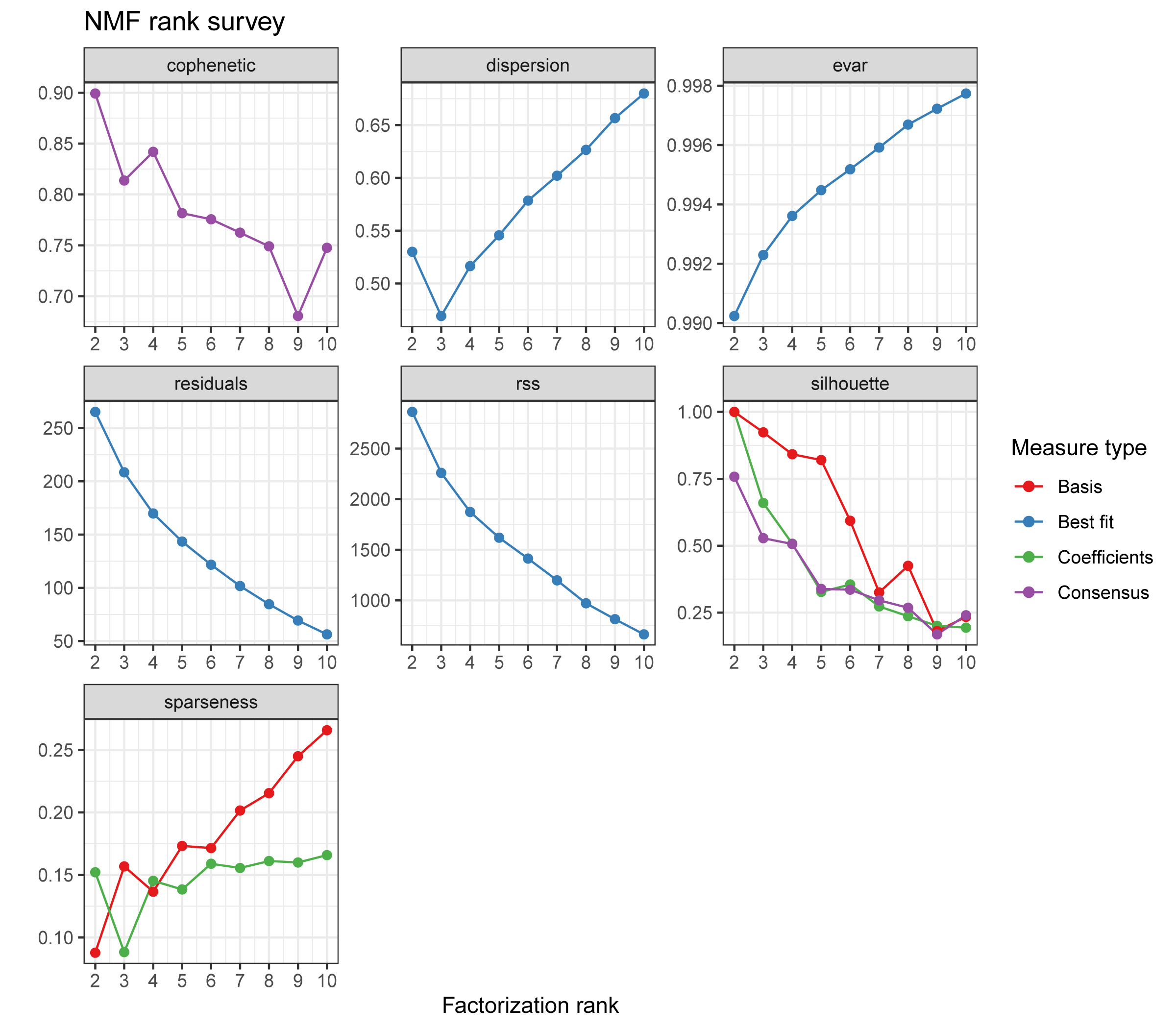

Supplement: Supplementary file 1 [file biomolecules-13-00736-s001.zip › Figure S1.jpg]

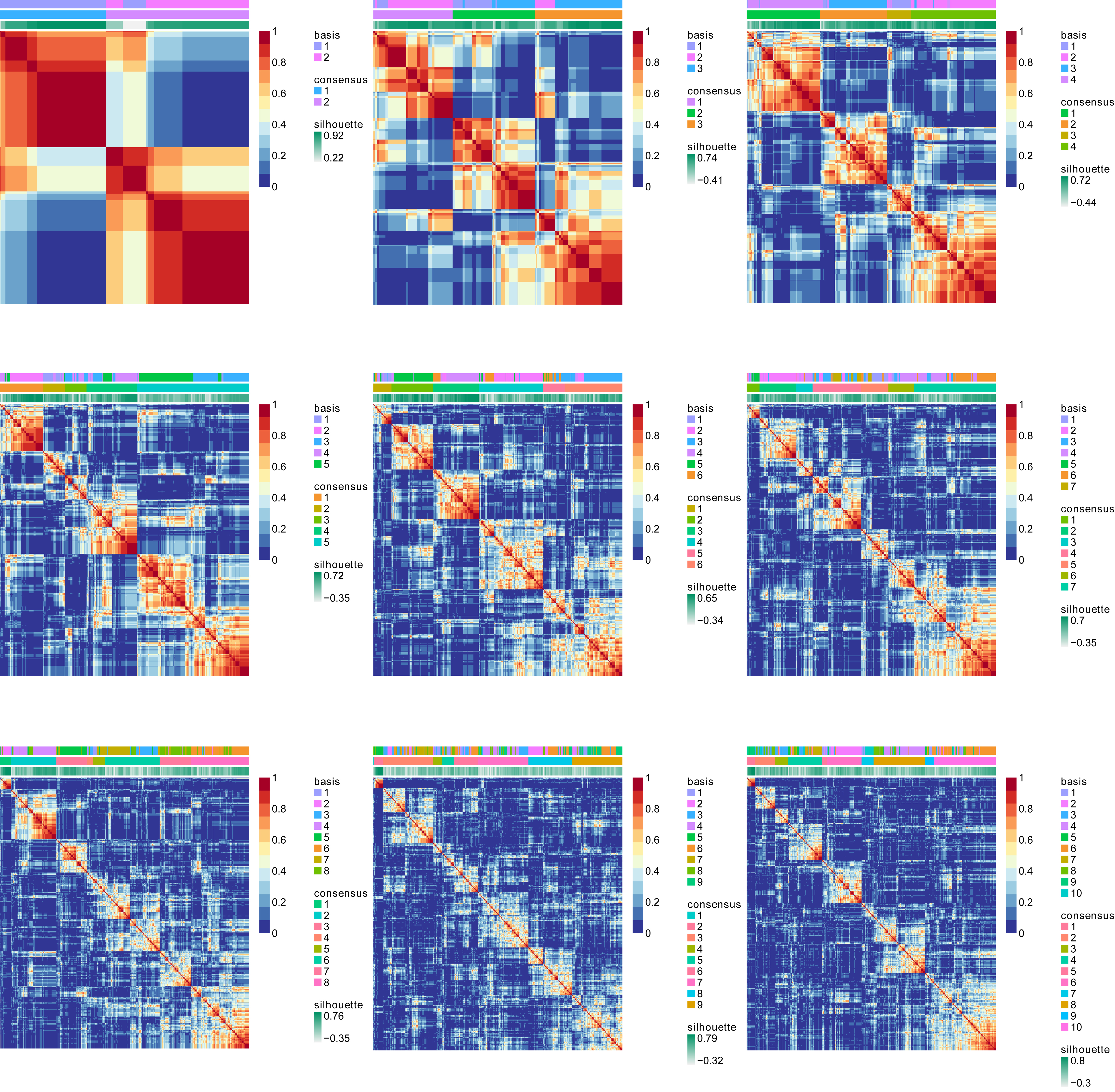

Supplement: Supplementary file 1 [file biomolecules-13-00736-s001.zip › Figure S2.jpg]

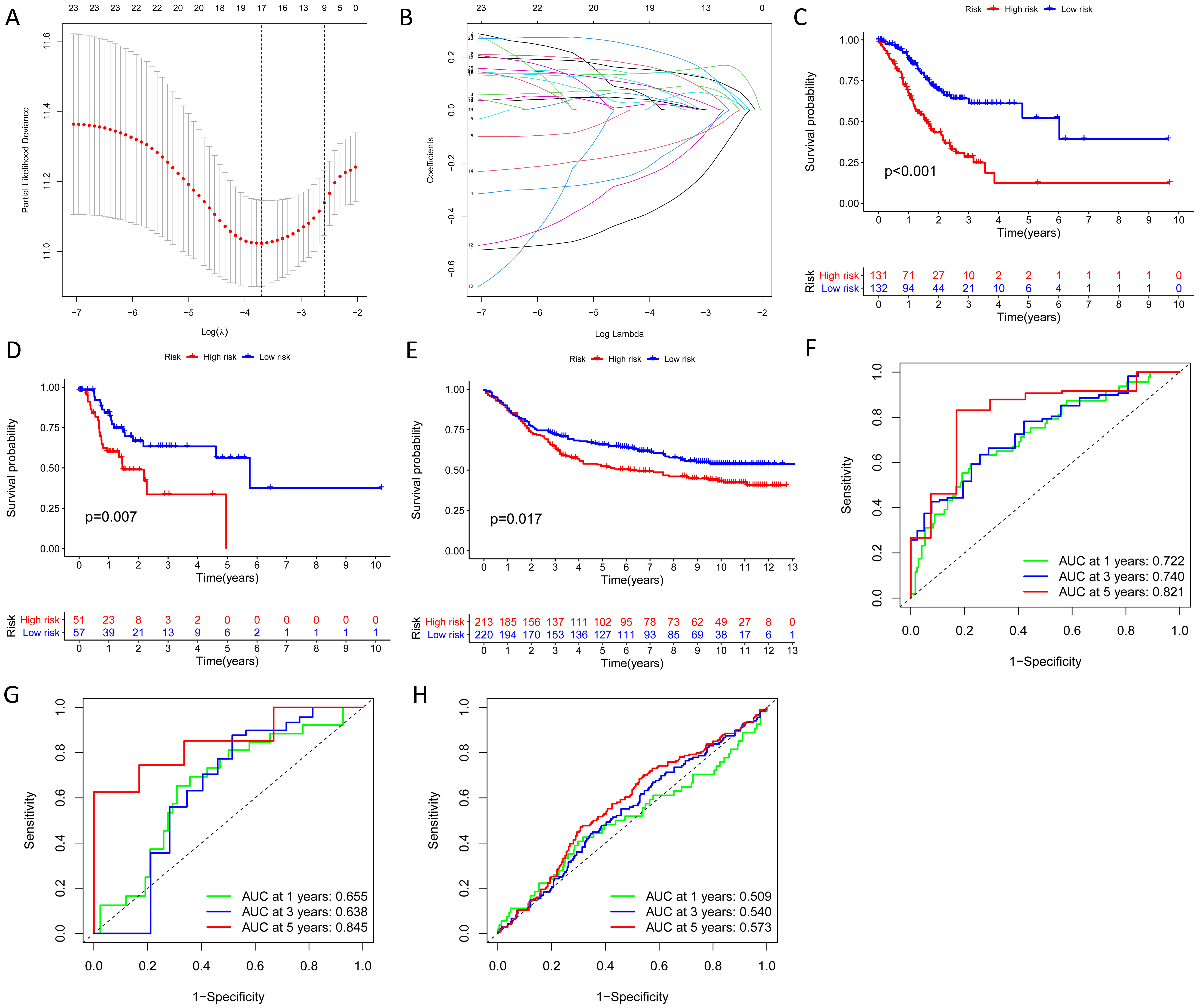

Supplement: Supplementary file 1 [file biomolecules-13-00736-s001.zip › Figure S3.jpg]

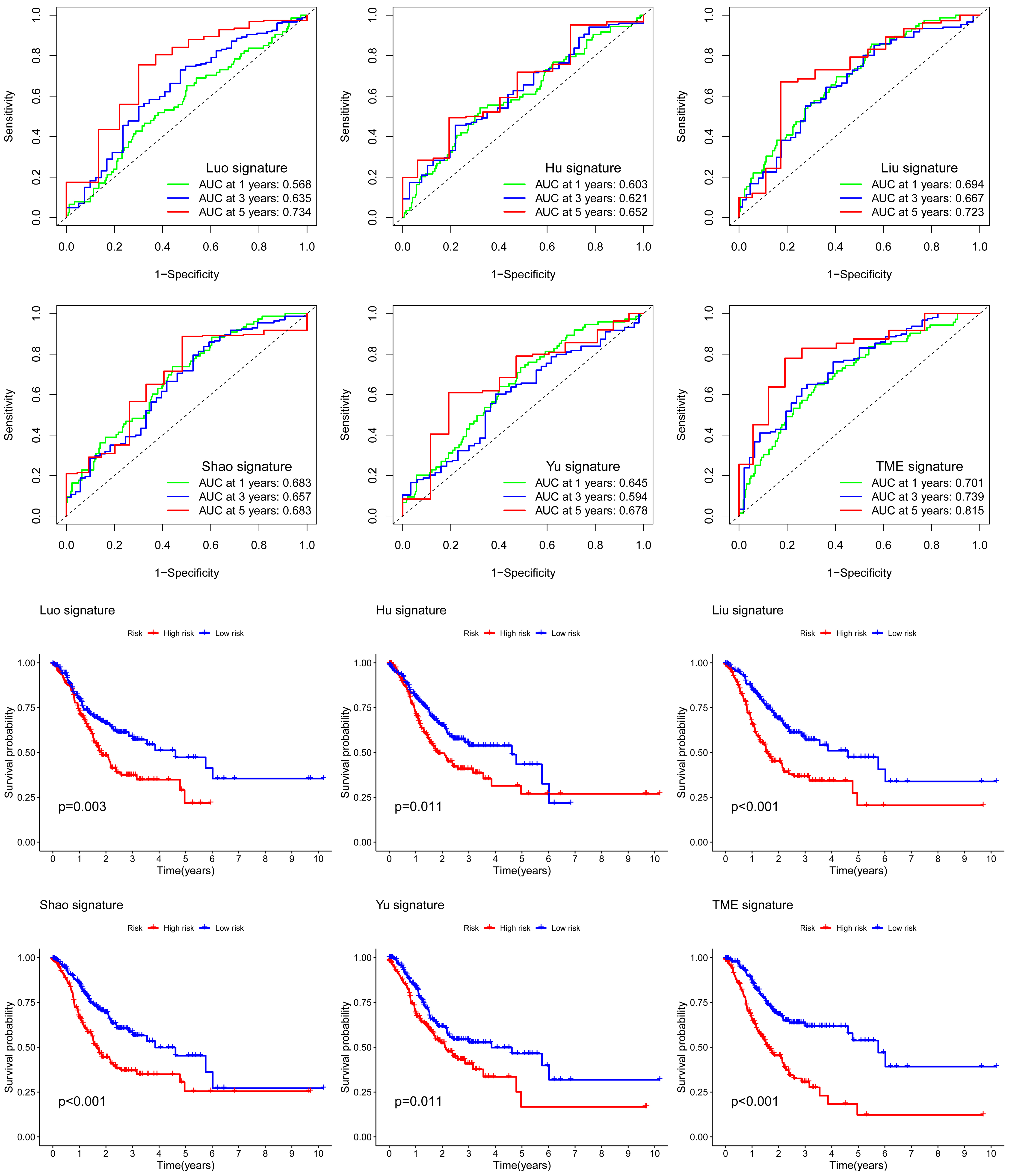

Supplement: Supplementary file 1 [file biomolecules-13-00736-s001.zip › Figure S4.jpg]

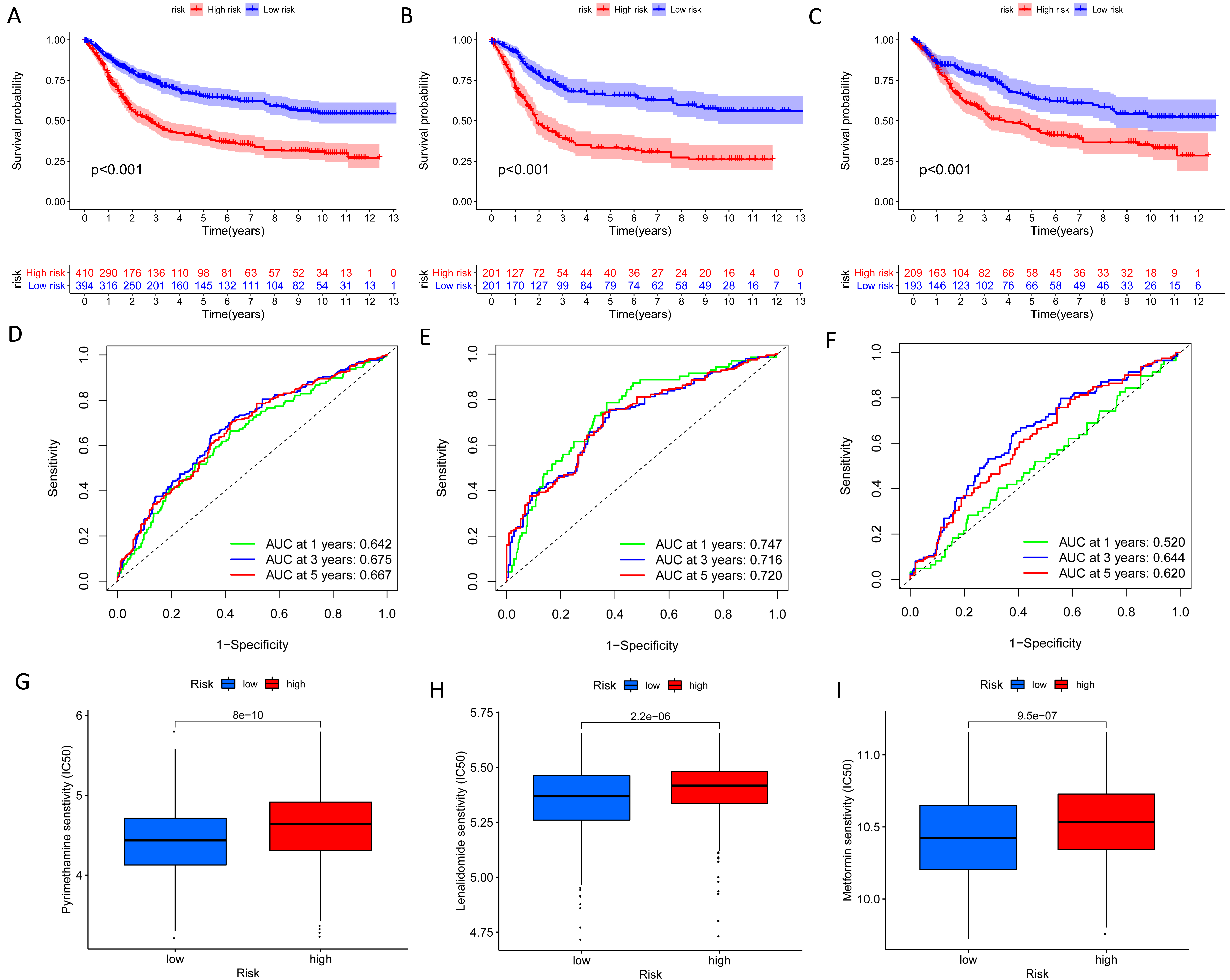

Supplement: Supplementary file 1 [file biomolecules-13-00736-s001.zip › Figure S5.jpg]

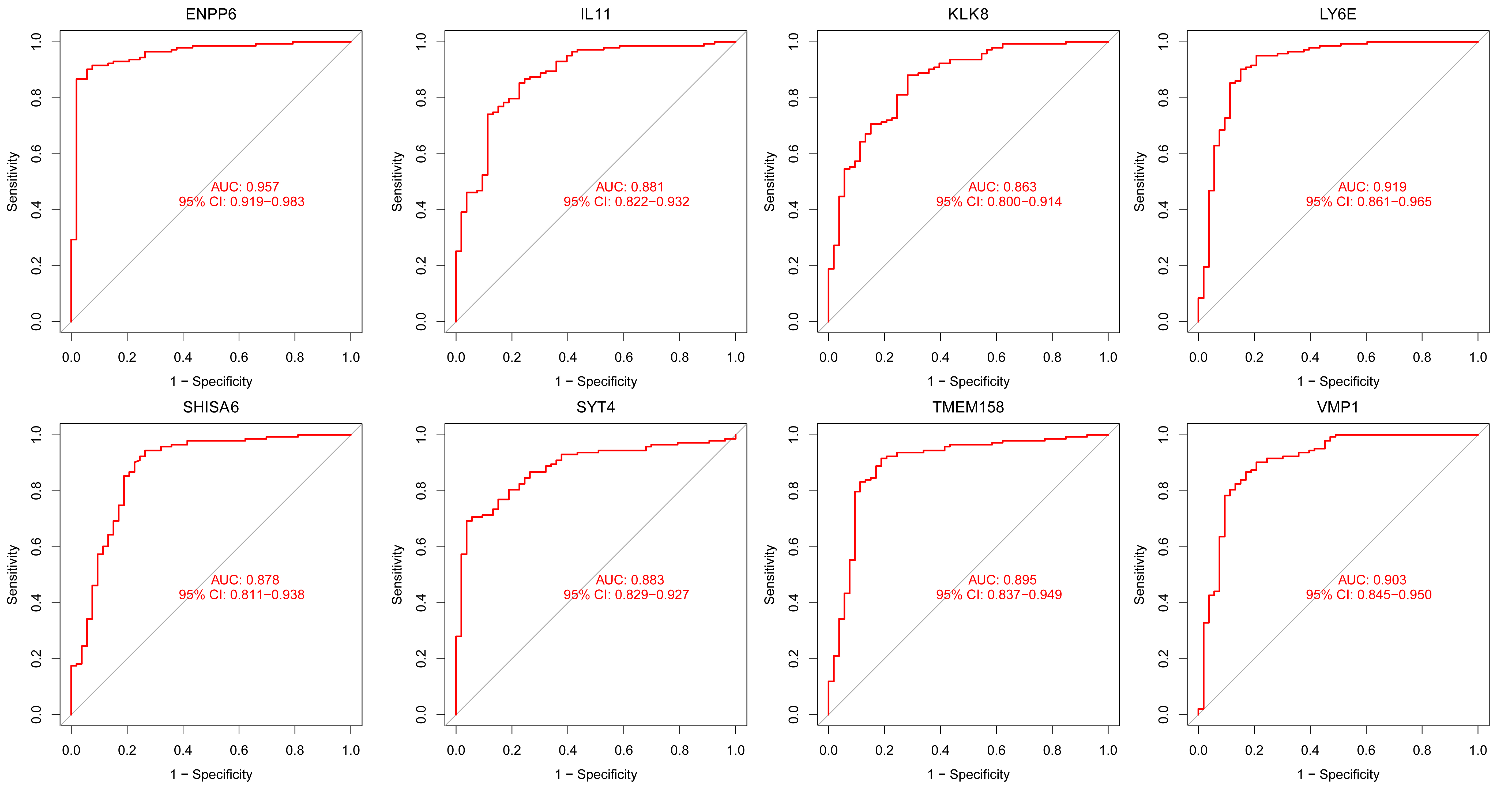

Supplement: Supplementary file 1 [file biomolecules-13-00736-s001.zip › Figure S6.jpg]

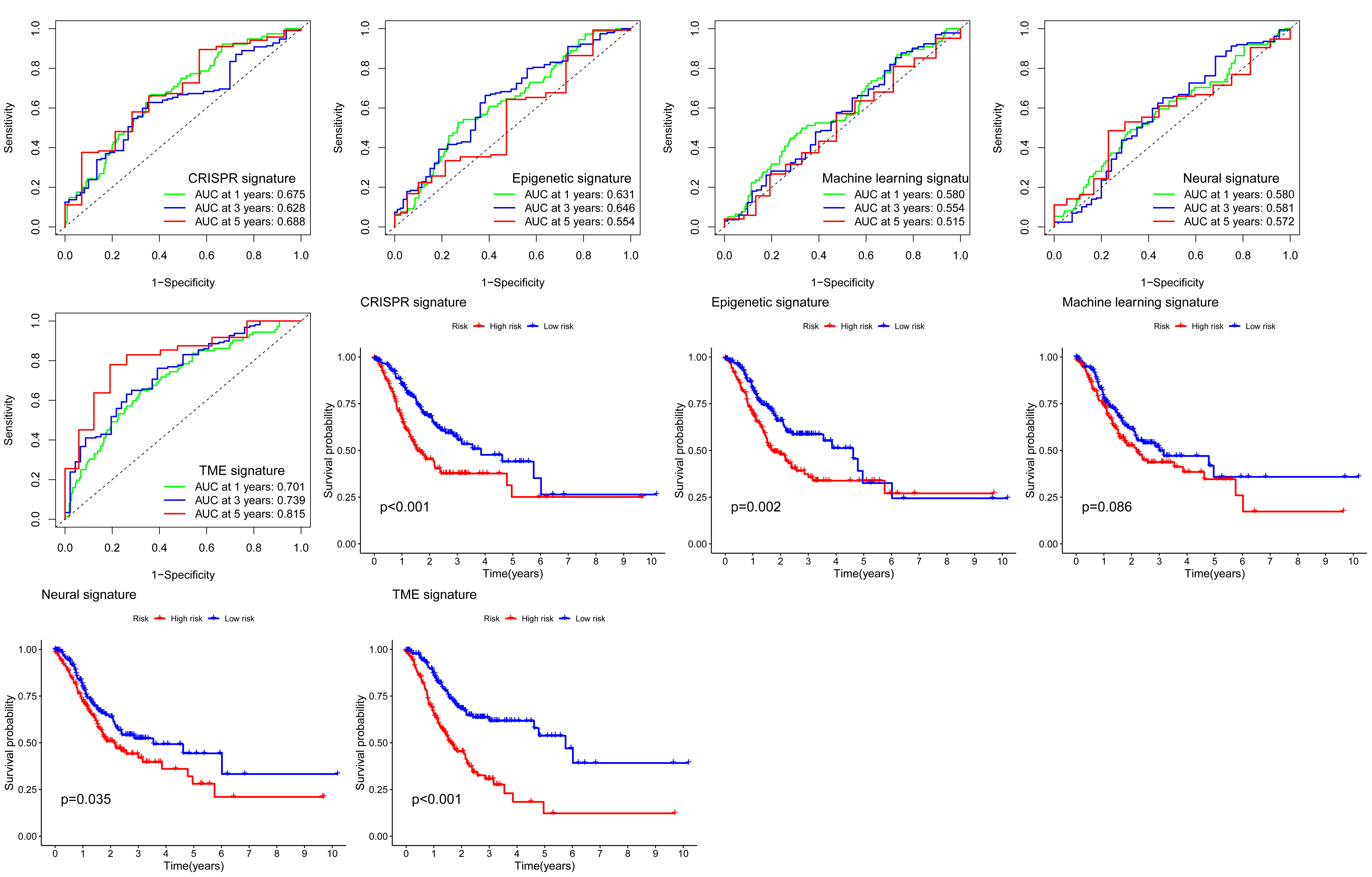

Supplement: Supplementary file 1 [file biomolecules-13-00736-s001.zip › Figure S7.jpg]

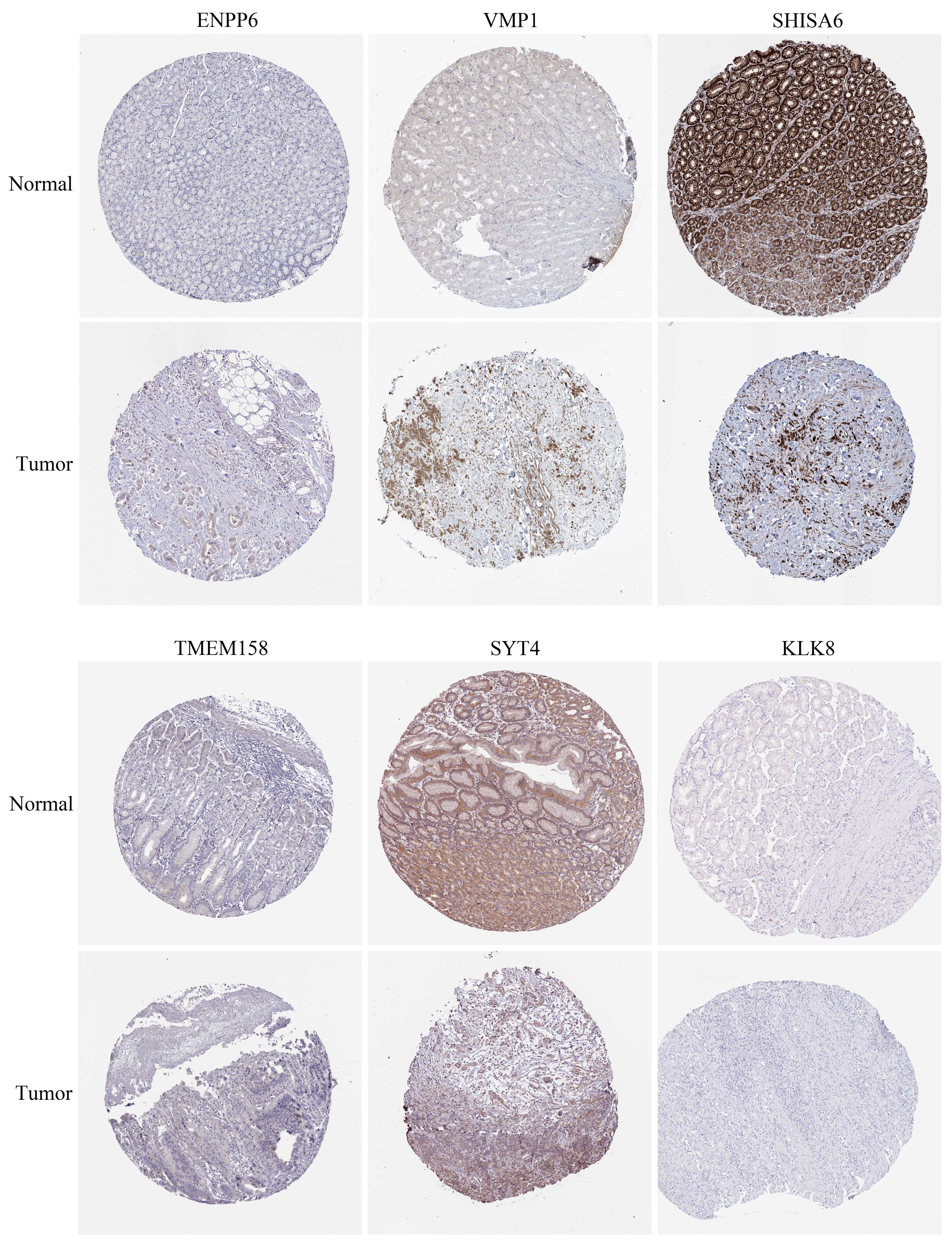

Supplement: Supplementary file 1 [file biomolecules-13-00736-s001.zip › Figure S8.jpg]
